# Plasma metabolites changes in male heroin addicts during acute and protracted withdrawal

**DOI:** 10.18632/aging.203311

**Published:** 2021-07-19

**Authors:** Yong Zhou, Zhenrong Xie, Zunyue Zhang, Jiqing Yang, Minghui Chen, Fengrong Chen, Yuru Ma, Cheng Chen, Qingyan Peng, Lei Zou, Jianyuan Gao, Yu Xu, Yiqun Kuang, Mei Zhu, Dingyun You, Juehua Yu, Kunhua Wang

**Affiliations:** 1 NHC Key Laboratory of Drug Addiction Medicine, Kunming Medical University, First Affiliated Hospital of Kunming Medical University, Kunming 650032, Yunnan, China; 2 Centre for Experimental Studies and Research, First Affiliated Hospital of Kunming Medical University, Kunming 650032, Yunnan, China; 3 Medical School, Kunming University of Science and Technology, Kunming 650032, Yunnan, China; 4 Yunnan Institute of Digestive Disease, First Affiliated Hospital of Kunming Medical University, Kunming 650032, Yunnan, China; 5 School of Public Health, Kunming Medical University, Kunming 650032, Yunnan, China; 6 Yunnan University, Kunming 650032, Yunnan, China

**Keywords:** heroin withdrawal, metabolomics, polyunsaturated fatty acids, aromatic amino acids, tricarboxylic acid cycle

## Abstract

Background: Heroin addiction and withdrawal have been associated with an increased risk for infectious diseases and psychological complications. However, the changes of metabolites in heroin addicts during withdrawal remain largely unknown.

Methods: A total of 50 participants including 20 heroin addicts with acute abstinence stage, 15 with protracted abstinence stage and 15 healthy controls, were recruited. We performed metabolic profiling of plasma samples based on ultraperformance liquid chromatography coupled to tandem mass spectrometry to explore the potential biomarkers and mechanisms of heroin withdrawal.

Results: Among the metabolites analyzed, omega-6 polyunsaturated fatty acids (linoleic acid, dihomo-gamma-linolenic acid, arachidonic acid, n-6 docosapentaenoic acid), omega-3 polyunsaturated fatty acids (docosahexaenoic acid, docosapentaenoic acid), aromatic amino acids (phenylalanine, tyrosine, tryptophan), and intermediates of the tricarboxylic acid cycle (oxoglutaric acid, isocitric acid) were significantly reduced during acute heroin withdrawal. Although majority of the metabolite changes could recover after months of withdrawal, the levels of alpha-aminobutyric acid, alloisoleucine, ketoleucine, and oxalic acid do not recover.

Conclusions: In conclusion, the plasma metabolites undergo tremendous changes during heroin withdrawal. Through metabolomic analysis, we have identified links between a framework of metabolic perturbations and withdrawal stages in heroin addicts.

## INTRODUCTION

Heroin, an illegal, highly addictive drug, is extremely harmful to human physical and mental health [1, 2]. Heroin addiction is defined as a chronic obsessive-compulsive brain disease, which causes overwhelming desire, increased tolerance, and severe withdrawal symptoms [3]. Major withdrawal symptoms include restlessness, insomnia, drug craving, diarrhea, muscle and bone pain, cold flashes with goose bumps, and leg movements [4]. Usually, the withdrawal symptoms peak within 8 to 72 hours after the last heroin dose and last for 4 to 10 days [4, 5]. However, some people may experience withdrawal symptoms for months or even years [6]. In particular, neurological/psychiatric symptoms of depression, anxiety, and cognitive impairment are extremely harmful to physical and mental health and are key factors that lead to addiction and relapse in patients with heroin use disorder [3, 7].

Neuroimaging studies have shown that dysfunction of the nucleus accumbens functional network is present in long-term withdrawal (> 3 years) heroin-dependent individuals [8]. Compared with the healthy control group, their gray matter volume around the parieto-occipital sulcus was significantly reduced [9]. The underlying mechanism of these dramatic changes after heroin withdrawal remains unclear. Studies have shown that drug addiction can be considered a metabolic disease; it is triggered by the destruction of the body’s metabolism and thereafter leads to persistent neurochemical disorders [10]. Therefore, it is particularly important to identify the causes of neurological/psychiatric symptoms after heroin withdrawal from the perspective of metabolism. However, previous metabolic studies related to drug addiction have mainly used animal studies [11–13] and human metabolism might be different from that of animal models. Understanding the status of the metabolic changes of heroin addicts is essential to address withdrawal symptoms.

Increasing data indicate that the metabolic changes of fatty acids (FAs) are involved in a variety of neurodegenerative diseases and neuropsychiatric diseases [14–17]. FAs are closely related to the neurogenesis of normal brain development, neuronal inflammation, and neurotransmitter production [18]. The polyunsaturated FAs (PUFAs) in particular are very important, because they play a key role in the brain with regard to neuron survival, neurogenesis, neurodegeneration, and aging [19]. For example, compared with a control group, the postmortem brain tissue derived from the patients with moderate Alzheimer’s disease (AD) has shown higher levels of alpha-linolenic acid [20]. Mood disorders are closely related to low eicosapentaenoic acid (EPA) and/or docosahexaenoic acid (DHA) status [21]. Amino acids (AAs) have become another focus of metabolomic studies because of their fundamental role in physiology. Series of biomarkers of AA metabolites have been found in many diseases. The combination of glutamine, glycine, and ornithine may serve as a potential diagnostic biomarker for autism spectrum disorder [22]. Single-voxel proton magnetic resonance spectroscopy has detected significant decreases in glutamate and glutamine within the medial frontal cortex in patients with major depression disorder compared with healthy controls [23]. Research on rat models found that methamphetamine and cocaine can cause changes in energy metabolism [11–13], suggesting that organic acids (OAs) play an important role in substance-related research. Taken together, the three types of metabolites (FAs, AAs, and OAs) may play important roles in neuropsychiatric diseases. However, there are few studies on human blood metabolism related to heroin addiction and withdrawal, and the related metabolic biomarkers have not been developed and utilized.

The advances in metabolomics provide powerful tools for profiling global biochemical changes in disease and treatment, which simultaneously identify and quantify hundreds to thousands of metabolites, providing an excellent opportunity to profile the metabolic changes in a high-throughput manner. Although brain tissues or cerebrospinal fluid are ideal biological samples for research on neuropsychiatric disorders, they cannot be practically obtained because of ethical and safety concerns. In comparison, blood samples can be acquired at minimal risk and cost. Furthermore, because the concentration of peripheral blood metabolites is closely related to pharmacological or toxicological effects, and because it can reflect the current state of the organism, metabolites in the peripheral blood are increasingly used to discover disease biomarkers [24, 25]. In a cohort study of schizophrenia, myriad differential metabolites were found in the peripheral blood of patients; these metabolites have potential to develop diagnostic tool [26, 27]. In this present study, we utilized an ultraperformance liquid chromatography coupled to tandem mass spectrometry (UPLC-MS/MS) metabolomics platform to determine the plasma metabolite profiles of heroin addicts currently undergoing withdrawal and with different withdrawal times. In particular, we focused on AAs, FAs, and OAs. This research will clarify the metabolic changes that occur in heroin addicts at different stages of withdrawal, which may generate valuable biomarkers of heroin withdrawal and change our view of acute heroin withdrawal.

## RESULTS

### Baseline characteristics

A total of 35 male heroin addicts including 20 with acute abstinence stage (ABS) and 15 with protracted abstinence stage (PABS), as well as 15 healthy controls (HCs), were recruited and analyzed in this work. The demographic characteristics of the participants are presented in Table 1. There were no significant differences in age, body mass index, education, smoking history, alcohol use, or tea drinking habits among the three groups of participants.

**Table 1:** Demographic features of drug withdrawal groups and HCs.

**Characteristics**	**ABS**	**PABS**	**HCs**	**FDR** **ABS vs. HC**	**FDR** **PABS vs. HC**	**FDR** **ABS vs. PABS**
Male	20	15	15	NA	NA	NA
Age, years^b^	32.9 ± 7.28	33.33 ± 5.34	34.53 ± 7.63	0.7256	0.7256	0.7256
BMI^b^	22.11 ± 3.20	22.04 ± 2.32	22.91 ± 3.32	0.9867	0.9867	0.9867
Drug withdrawal, day^b^	3.1 ± 2.97	145.07 ± 12.98	NA	NA	NA	4.04E-7
Education, years^b^	7.37 ± 2.36	7.8 ± 2.65	10.71 ± 5.58	0.1681	0.2430	0.6479
History of heroin abuse, years^b^	7.45 ± 6.6	15.8 ± 7.65	NA	NA	NA	2.36E-3
Relapse, no. of times (1/2/3/5)^a^	12/7/0/1	8/2/4/1	NA	NA	NA	0.0523
Manner of drug use (snorting/injection)^a^	16/4	10/5	NA	NA	NA	0.6154
Smoking history (Y/N)^a^	20/0	15/0	14/1	1.0000	1.0000	1.0000
Drinking history (Y/N)^a^	6/14	6/9	3/12	0.3183	0.6384	1.0000
Tea drinking history (Y/N)^a^	3/17	5/10	3/12	1.0000	1.0000	1.0000

Continuous variables are expressed as mean ± standard deviation.

Abbreviations: BMI, body mass index; FDR, false discovery rate; HCs, healthy controls; NA, not available.

^a^Analyzed by the Fisher test or chi squared test.

^b^Analyzed by the Wilcoxon test.

### Overview of the plasma metabolites

**Figure 1:**
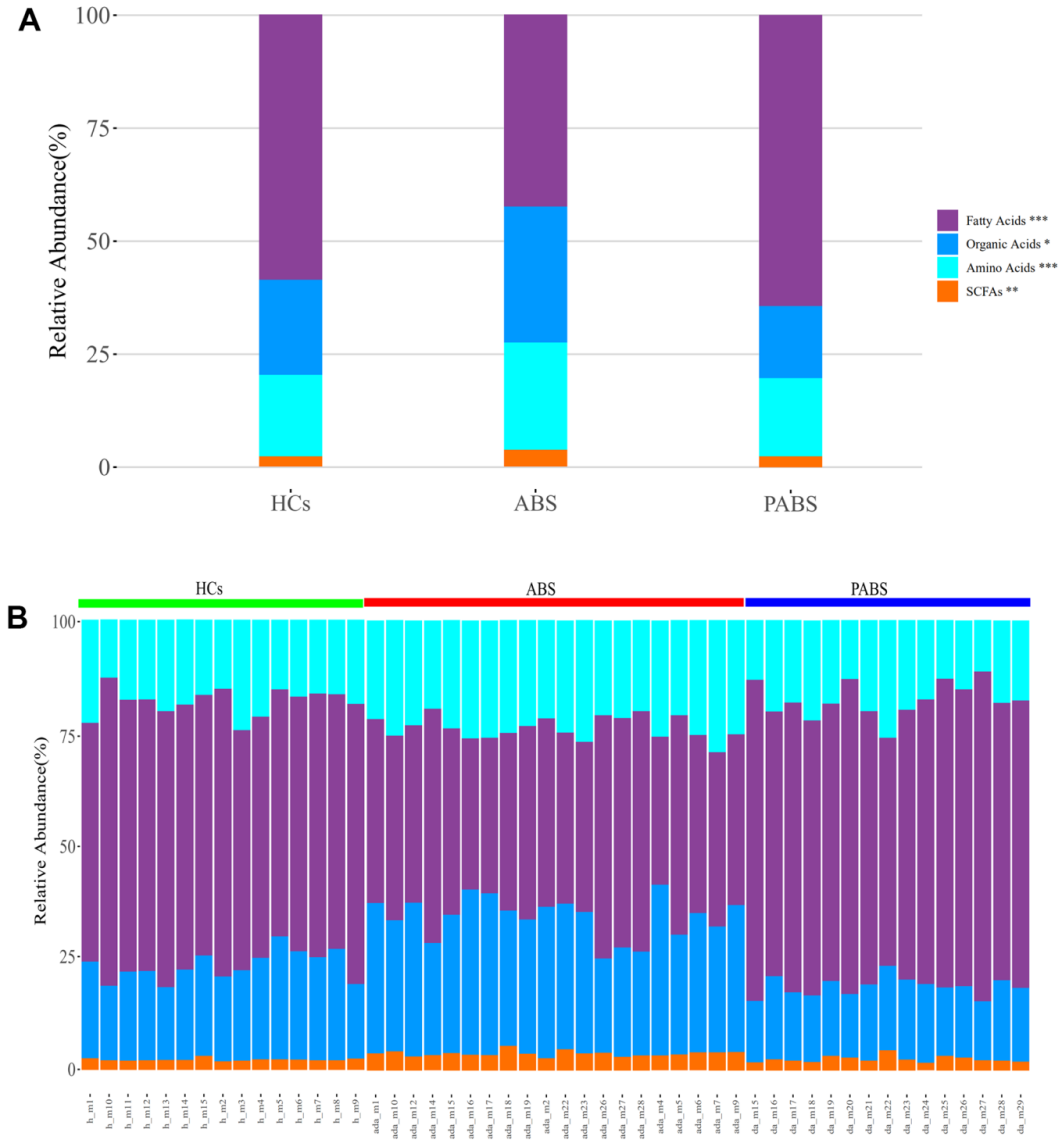
**Heroin withdrawal changes the overall composition of metabolites in plasma.** (**A**) The relative abundance of the four types of metabolites in each group. (**B**) The relative abundance of the four types of metabolites in each sample. ^*^P<0.05, ^**^P <0.01, ^***^P<0.001 (kruskal.test). Results are presented as means ± SE.

Using UPLC-MS/MS, 129 endogenous plasma metabolites (metabolites naturally produced by an organism, including fatty acids, amino acids, organic acids, nucleic acids, amines, sugars, vitamins, co-factors, pigments, antibiotics, etc.) were identified, including 43 AAs, 51 FAs, eight short-chain FAs (SCFAs), and 27 OAs (Supplementary Table 1). The overall composition of the four types of metabolites from plasma samples of each group significantly differed (Figure 1). The relative abundances of FAs, OAs, AAs, and SCFAs in HCs were 58.7%, 20.9%, 18.1%, and 2.3%, respectively. Conversely, the relative abundances of FAs, OAs, AAs, and SCFAs in ABS were 42.5%, 30%, 23.7%, and 3.8%, respectively, and were 64.3%, 15.9%, 17.3%, 2.5%, respectively in PABS. Notably, the relative abundance of FAs in ABS was lower than in the other two groups, whereas the relative abundances of OAs, AAs, and SCFAs were higher than in the other two groups.

### Differences in metabolites between ABS and the other two groups

**Figure 2:**
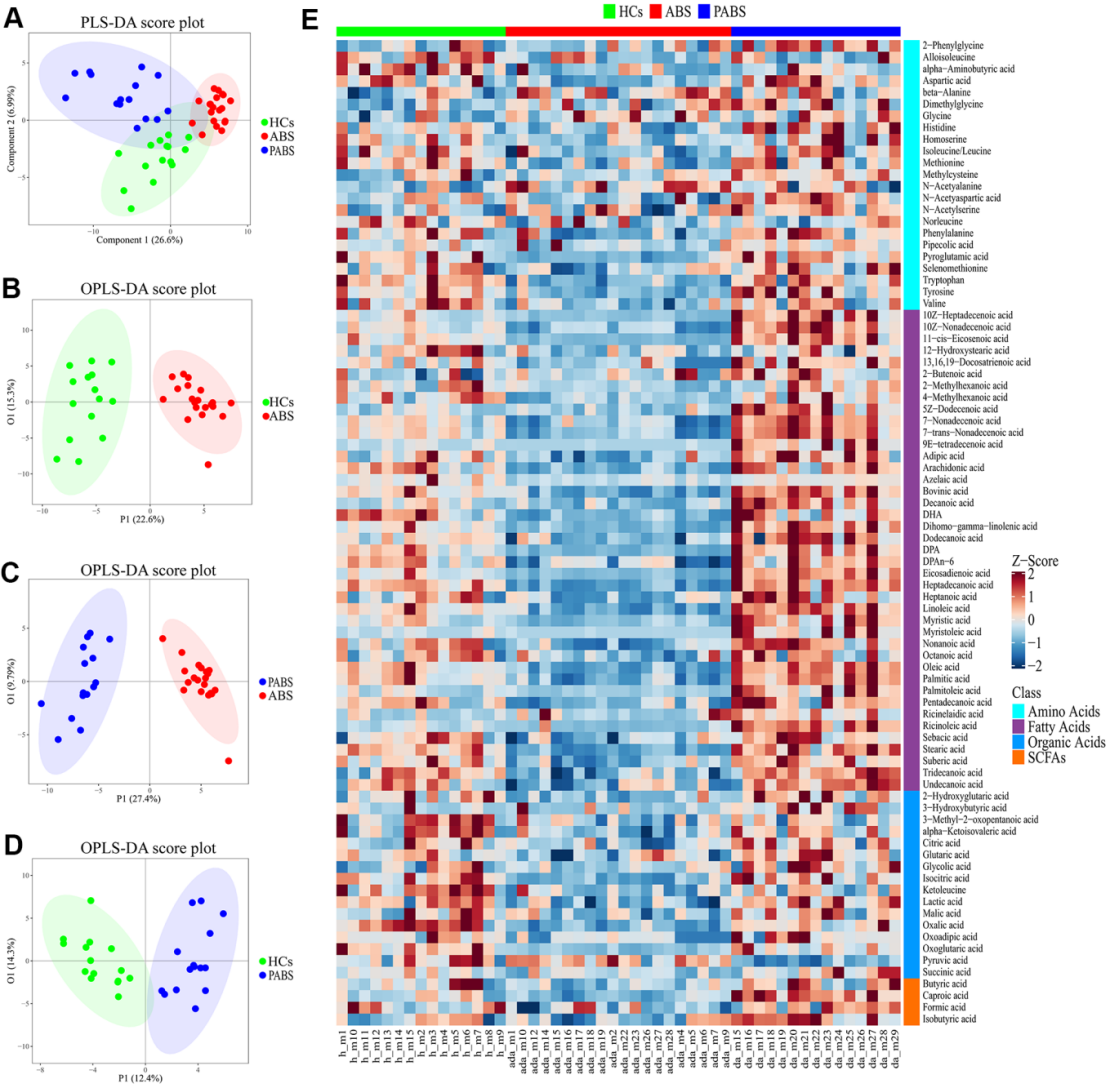
**Obvious metabolic abnormalities during acute heroin withdrawal.** (**A**) PLS-DA score plot of plasma samples obtained from the 3 groups. (**B**) OPLS-DA score plot showing separation by phenotype between healthy controls and acute heroin withdrawal. (**C**) OPLS-DA score plot showing separation by phenotype between long-term heroin withdrawal and acute heroin withdrawal. (**D**) OPLS-DA scores plot showing separation by phenotype between healthy controls and long-term heroin withdrawal. (**E**) The heatmap of differential metabolites between the 3 groups.

Multivariate statistical analysis was performed to compare the differences in plasma metabolic profiling among the three groups. As shown in the score plot of PLS-DA (Figure 2A), a clear separation was observed among these three groups, suggesting that the overall metabolic pattern was altered in the plasma after heroin withdrawal and that the length of heroin withdrawal time may affect the metabolism. To distinguish in more detail the differences in metabolic profiles among the groups, a supervised OPLS-DA model was carried out between group pairs (Figure 2B–2D). Furthermore, the variables were unit variance scaled, and cross-validation with 1,000-times permutation tests were used to identify the reliability of the models. The R2Y and Q2Y values of the OPLS-DA model for ABS versus HC groups were 0.92 and 0.869, respectively, with two components responsible for the classification. The R2Y and Q2Y values of the OPLS-DA model for PABS versus ABS were 0.939 and 0.79, respectively, with two components. Moreover, the R2Y and Q2Y values of the OPLS-DA model for PABS versus HCs were 0.872 and 0.639, respectively, with two components (Supplementary Figure 1). According to the normality of the data and the homogeneity of variance, we selected the univariate ANOVA test or the Kruskal-Wallis test (p < 0.05) to measure the difference in metabolites among the three groups. The heat plot for the differential metabolites in ABS versus PABS versus HCs is presented in Figure 2E. Most of these differential metabolites were significantly lower in ABS, and they could be restored during the next few months.

### Effect of the heroin withdrawal on metabolite levels

**Figure 3:**
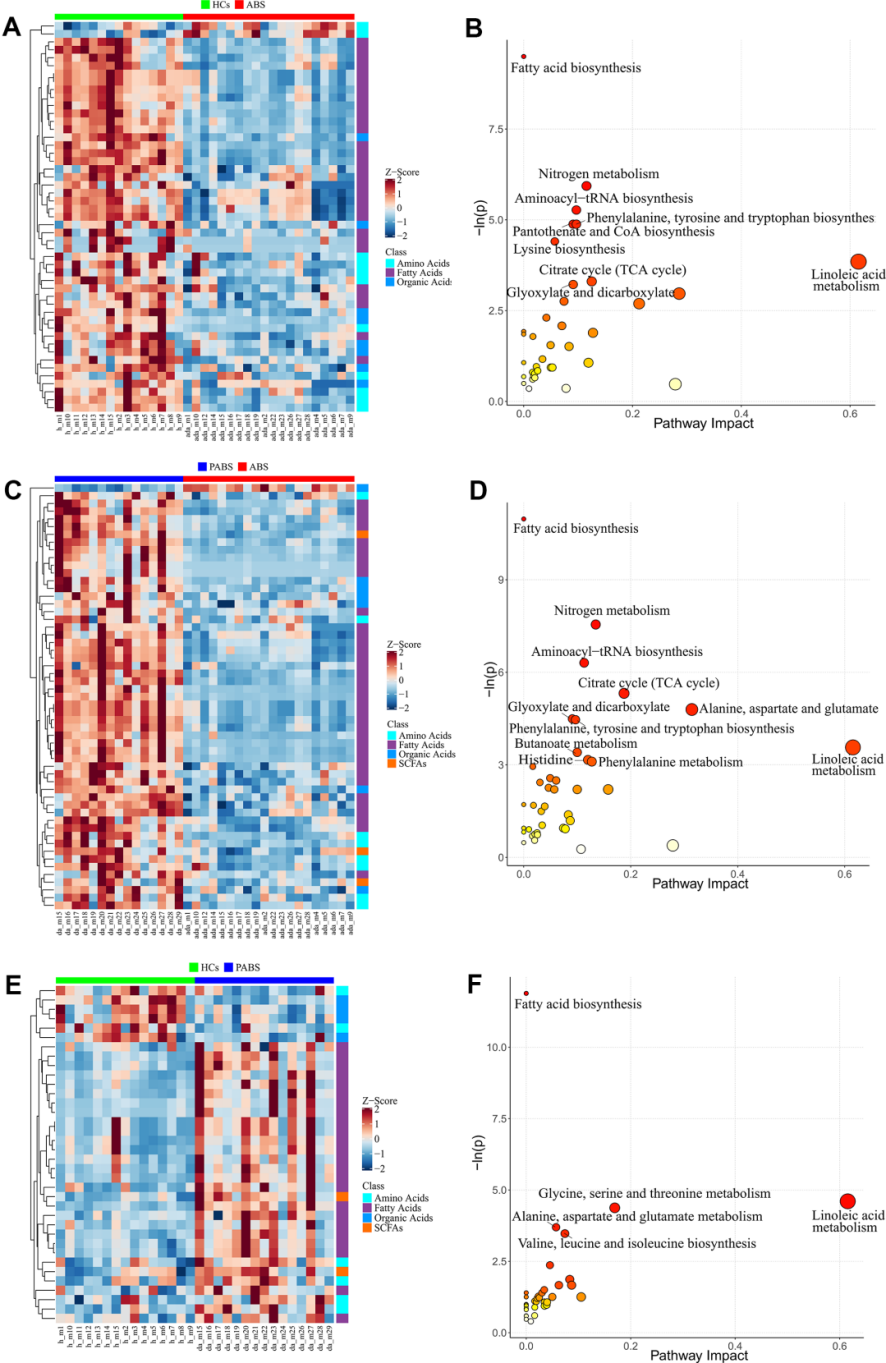
**Differential metabolites and pathway enrichment analysis between each two groups.** (**A**, **B**) The heatmap and pathway impact analysis of differential metabolites between healthy controls and acute heroin withdrawal. (**C**, **D**) The heatmap and pathway impact analysis of differential metabolites between long-term heroin withdrawal and acute heroin withdrawal. (**E**, **F**) The heatmap and pathway impact analysis of differential metabolites between healthy controls and long-term heroin withdrawal. Note that pathway with P<0.05 will be marked with names in the figure.

**Figure 4:**
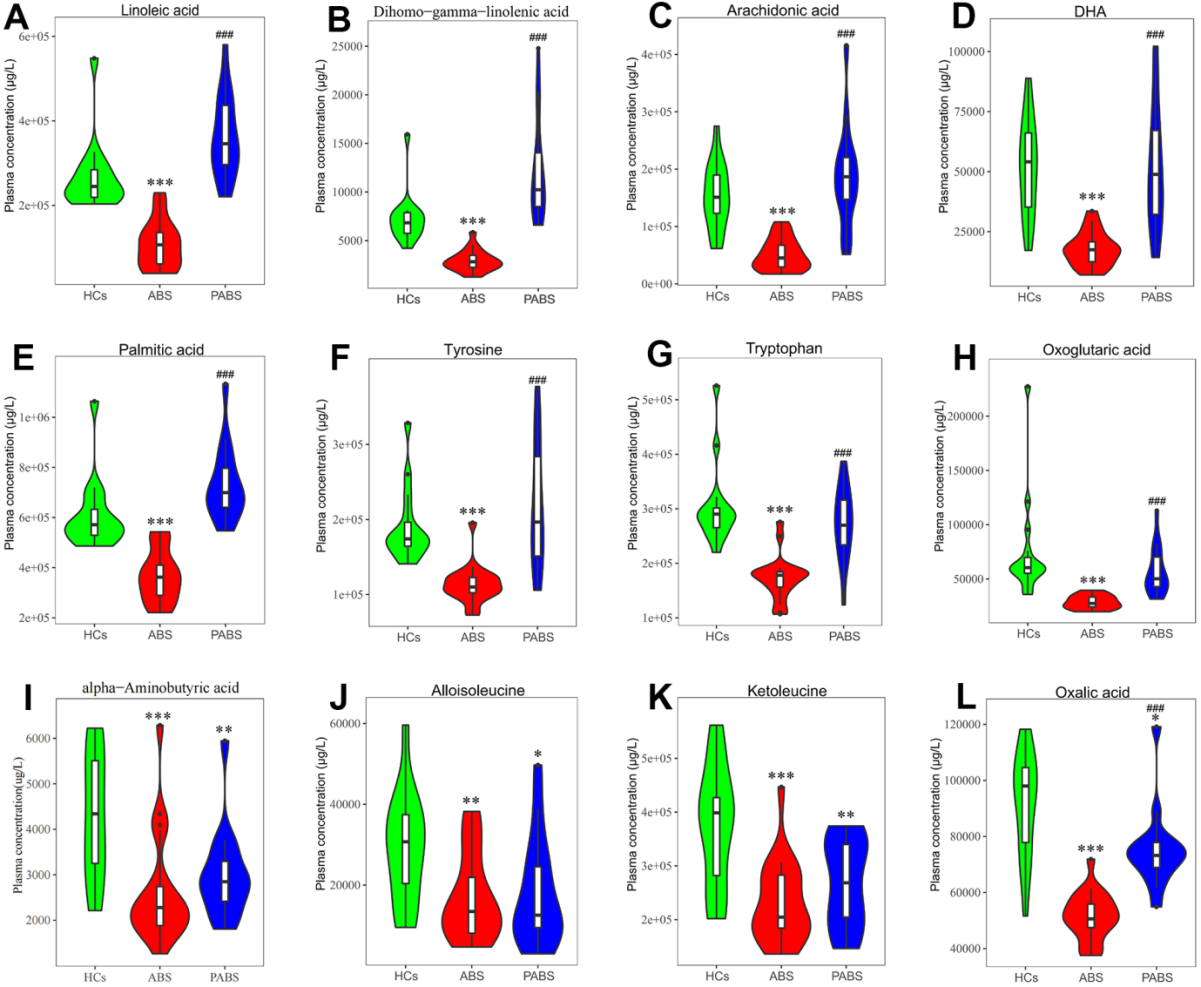
**Concentrations of plasma metabolites at 3 different stages.** (**A**–**D**) omega-6 and omega-3 polyunsaturated fatty acids. (**E**–**H**) representative fatty acid, amino acids and organic acid. (**I**–**L**) metabolites that have not recovered after long-term heroin withdrawal (^*^P < 0.05, ^**^P < 0.01, ^***^P < 0.001, compared with healthy controls. ^#^P < 0.05, ^##^P < 0.01, ^###^P < 0.001, compared with acute heroin withdrawal). Note: dihomo-gamma-linolenic acid is the polyunsaturated fatty acid with the most significant difference, tyrosine is the neurotransmitter precursor with the most significant difference, and oxoglutaric acid is the organic acid with the most significant difference in energy metabolism.

To obtain additional data about the differential metabolites of each group, we adopted univariate and multi-dimensional statistical methods and screened out the differential metabolites that met the double standard. These metabolites were the most reliable differential markers and may become potential biomarkers. The final list of screened differential metabolites, which met the set threshold (VIP ≥ 1 and p < 0.05), are displayed in Supplementary Table 2. Overall, 49 different metabolites were screened between HCs and ABS; 55 different metabolites were screened between ABS and PABS; and 36 different metabolites were screened between HCs and PABS (Figure 3A, 3C, 3E). Among these different metabolites, linoleic acid (LA), dihomo-gamma-linolenic acid, arachidonic acid, and n-6 docosapentaenoic acid (DPA) belong to the omega-6 (i.e., n-6) PUFAs; DHA and DPA belong to the omega-3 (i.e., n-3) PUFAs. These n-6 and n-3 PUFAs were significantly decreased in ABS compared with the HC group and were significantly increased in PABS (Figure 4A–4D). The same trend also appeared with other FAs, AAs, and OAs (Figure 4E–4H). Although most differential metabolites returned to normal after an extended withdrawal time, four kinds of metabolites (alpha-aminobutyric acid, alloisoleucine, ketoleucine, and oxalic acid) did not have normal levels restored even during protracted withdrawal several months of withdrawal (Figure 4I–4L).

### Alterations in metabolic pathways

We developed a pathway analysis bubble plot based on different metabolite results obtained from comparison of two group each, combined with pathway enrichment analysis and pathway topology analysis to facilitate biological interpretation and thereby reveal the most relevant pathways involved in heroin withdrawal (Figure 3B, 3D, 3F). The metabolites enriched in the pathway can be seen in Tables 2–4. By enriching the different metabolites between groups, nine significantly altered pathways (p < 0.05) were enriched between HCs and ABS; 11 significantly altered pathways were enriched between ABS and PABS; and only five significantly altered pathways were enriched between HCs and PABS. More importantly, seven pathways in the first two enrichment sets were identical. These were FA biosynthesis; nitrogen metabolism; aminoacyl-tRNA biosynthesis; citrate cycle (tricarboxylic acid [TCA] cycle); glyoxylate and dicarboxylate metabolism; phenylalanine, tyrosine, and tryptophan biosynthesis; and LA metabolism. In the three-pathway analyses of differential metabolite enrichment, two pathways (FA biosynthesis, LA metabolism) always existed. Interestingly, compared with HCs, the differential metabolites contained in the two pathways (myristic acid, dodecanoic acid, palmitoleic acid, stearic acid, oleic acid, palmitic acid, LA, and bovinic acid) were low in ABS and significantly increased in PABS. In addition, the pathways significantly affected by acute heroin withdrawal were nitrogen metabolism, aminoacyl-tRNA biosynthesis, phenylalanine, tyrosine and tryptophan biosynthesis, the citrate cycle (TCA cycle), and glyoxylic acid and dicarboxylic acid metabolism.

**Table 2:** Significantly altered pathways between the HCs (n = 15) and ABS (n = 20).

**Pathways**	**HC vs. ABS**		
	**p**	**Upregulated**	**Downregulated**
Fatty acid biosynthesis	< 0.001		Myristic acid Dodecanoic acid Palmitoleic acid Stearic acid Oleic acid Palmitic acid
Nitrogen metabolism	0.003		Phenylalanine Tyrosine Tryptophan Aspartic acid
Aminoacyl-tRNA biosynthesis	0.005		Phenylalanine Aspartic acid Methionine Tryptophan Tyrosine
Pantothenate and CoA biosynthesis	0.008	beta-Alanine	alpha-Ketoisovaleric acid Aspartic acid
Phenylalanine, tyrosine, and tryptophan biosynthesis	0.008		Phenylalanine Tyrosine Tryptophan
Lysine biosynthesis	0.012		Aspartic acid Oxoglutaric acid Oxoadipic acid
Linoleic acid metabolism	0.021		Linoleic acid Bovinic acid
Citrate cycle (TCA cycle)	0.037		Oxoglutaric acid Isocitric acid
Glyoxylate and dicarboxylate metabolism	0.040		Oxoglutaric acid Isocitric acid Oxalic acid

Abbreviations: ABS, abstinence syndrome; HC, healthy controls; TCA, tricarboxylic acid.

p < 0.05 is significant.

**Table 3:** Significantly altered pathways between PABS (n = 15) and ABS (n = 20).

**Pathways**	**PABS vs. ABS**		
	**p**	**Upregulated**	**Downregulated**
Fatty acid biosynthesis	1.72E-05		Myristic acid Dodecanoic acid Palmitoleic acid Decanoic acid Stearic acid Oleic acid Palmitic acid
Nitrogen metabolism	0.000527		Phenylalanine Tyrosine Tryptophan Aspartic acid Histidine
Aminoacyl-tRNA biosynthesis	0.001824		Histidine Phenylalanine Aspartic acid Methionine Tryptophan Tyrosine
Citrate cycle (TCA cycle)	0.0049	Pyruvic acid	Oxoglutaric acid Isocitric acid
Alanine, aspartate, and glutamate metabolism	0.008278	Pyruvic acid	Aspartic acid Oxoglutaric acid
Glyoxylate and dicarboxylate metabolism	0.011203	Pyruvic acid	Oxoglutaric acid Isocitric acid Oxalic acid
Phenylalanine, tyrosine, and tryptophan biosynthesis	0.011526		Phenylalanine Tyrosine Tryptophan
Linoleic acid metabolism	0.028252		Linoleic acid Bovinic acid
Butanoate metabolism	0.033159	Pyruvic acid	Butyric acid Oxoglutaric acid
Histidine metabolism	0.04232		Histidine Aspartic acid Oxoglutaric acid
Phenylalanine metabolism	0.04479	Pyruvic acid	Phenylalanine Tyrosine

Abbreviations: ABS, abstinence syndrome; PABS, post-abstinence syndrome; TCA, tricarboxylic acid.

p < 0.05 is significant.

**Table 4:** Significantly altered pathways between HCs (n = 15) and PABS (n = 15).

**Pathways**	**HCs vs. PABS**		
	**p**	**Upregulated**	**Downregulated**
Fatty acid biosynthesis	6.88E-06	Myristic acid Dodecanoic acid Palmitoleic acid Decanoic acid Oleic acid Palmitic acid	
Linoleic acid metabolism	0.010013	Linoleic acid Bovinic acid	
Glycine, serine, and threonine metabolism	0.012583	Dimethylglycine Glycine	Pyruvic acid
Alanine, aspartate, and glutamate metabolism	0.02486		N-Acetylaspartic acid Pyruvic acid
Valine, leucine, and isoleucine biosynthesis	0.031022		Pyruvic acid Ketoleucine

Abbreviations: HCs, healthy controls; PABS, post-abstinence syndrome.

p < 0.05 is significant.

## DISCUSSION

In this study, we implemented UPLC-MS/MS–based metabolomics approaches to investigate the alterations of metabolites in the peripheral blood of heroin addicts undergoing withdrawal in different stages. This study aimed to identify potential biomarkers and elucidate the possible mechanism involving heroin withdrawal from a metabolic perspective.

### Severe metabolic alterations of n-6 and n-3 PUFAs during acute heroin withdrawal

The clinical characteristics of depressive and anxiety symptoms are closely related to blood levels of PUFAs [28, 29]. In the study by Xie et al. [30], the levels of LA and arachidonic acid in the hair of heroin abusers were lower than those in the control group [30]. In our study, the consistent result appeared in ABS. LA is considered essential, because it cannot be synthesized in humans. LA can be converted into dihomo-gamma-linolenic acid, and then into arachidonic acid, in the body [29]. In cell membrane phospholipids, arachidonic acid is a substrate for the synthesis of many biologic compounds [30]. Therefore, the membrane content of arachidonic acid is very important. Changes in arachidonic acid can affect signal pathways inside and outside the cell and affect gene expression and physiological metabolic reactions, which in turn ultimately affects health status [29]. Although LA and arachidonic acid are the precursors of many effective pro-inflammatory mediators, such as prostaglandins and leukotrienes, they do not have pro-inflammatory effects. Instead, they have a wide range of physiological effects in a complex body [29, 31–33]. For example, supplementation of arachidonic acid normalize neurogenesis and behavior in depressed mice [34]. During acute heroin withdrawal, the significant reduction of n-6 PUFAs may seriously interfere with the signaling pathways both inside and outside the cell, leading to changes in mental or physical behaviors that may be closely related to painful withdrawal symptoms.

Emerging studies have shown that n-3 PUFAs support health and reduce the risk of chronic diseases [35, 36]. Low levels of n-3 PUFAs are associated with many different diseases, such as depression, anxiety, and AD [17, 37]. As a component of the cell membrane, n-3 PUFAs can affect the biosynthesis of eicosanoids by regulating the fluidity of the cell membrane or the complex assembly in lipid rafts, thereby affecting cell signal transduction and exerting immune regulation [36, 38]. In central nervous system, n-3 PUFAs are regulators of gene transcription, and they play a vital role in maintaining brain structure [39]. In addition, Supplementation of n-3 PUFAs has a positive regulatory effect on neuropsychiatric diseases. For example, DHA supplementation in people with mild cognitive impairment could significantly improve cognitive function and slow the progression of hippocampal atrophy [40]. The decreases of n-3 PUFAs (DHA and DPA) during acute heroin withdrawal indicate that heroin has caused long-lasting and serious damage to the body.

### Changes in aromatic AA metabolism during acute heroin withdrawal

Aromatic AAs include phenylalanine (Phe), tyrosine (Tyr), and tryptophan (Trp). Phe can be transformed into Tyr through phenylalanine hydroxylase, and Tyr could be metabolized further into neurotransmitters, such as dopamine, norepinephrine, epinephrine, and melanin, through the action of tyrosine hydroxylase. Trp could be metabolized through the kynurenine and serotonin pathways to produce biologically active compounds, such as serotonin, melatonin, and niacin. Heroin damages the reward system by affecting the activities of dopaminergic, gamma-aminobutyric acid (GABA), serotonergic, and cholinergic neurotransmitters in the central nervous system [41, 42]. As the precursor AAs of catecholamines, Phe and Tyr have a wide range of physiological effects. Metabolic disorders in Phe and Tyr can lead to neurodystrophy and depression [43, 44]. Trp is involved in several physiological processes, including neuronal function, immunity, and gut homeostasis [45]. Plasma Trp decreases occur in many diseases, such as major depression, schizophrenia, Parkinson’s disease, and AD [46–50]. Our results showed that the aromatic AAs are significantly reduced during acute heroin withdrawal. The reduction may have a great impact on the concentration of neurotransmitters in the central nervous system, especially some neurotransmitters that cannot directly pass through the blood-brain barrier, such as serotonin and dopamine.

### Significant changes in energy metabolism during acute heroin withdrawal

The TCA cycle is carried out in the mitochondrial matrix of the human body. It is the final metabolic pathway of the three main nutrients (carbohydrates, lipids, and AAs) and is the hub of energy metabolism. Our results suggest that, during acute heroin withdrawal, oxoglutaric acid and isocitric acid are significantly reduced. The two are important intermediate products of the TCA cycle. The synthesis of oxoglutaric acid from isocitric acid is an important rate-limiting step in the TCA cycle. The reduction of these metabolites indicates that the TCA cycle is largely affected, mitochondrial function is impaired, energy metabolism is weakened, and productivity is reduced during heroin withdrawal. This finding suggests that the TCA cycle is downregulated during acute heroin withdrawal. However, the results of animal experiments are contrary to this. Results from Zheng et al. [51] show that, in rats with heroin withdrawal for 4 days, serum citric acid (a TCA cycle intermediate) remains elevated, indicating upregulation of the TCA cycle. Oxoglutaric acid helps stimulate collagen synthesis and can affect age-related processes, including stem cell proliferation, and it has emerged as master regulatory metabolite in aging biology [52, 53]. The decrease of oxoglutaric acid during the acute withdrawal also suggests that heroin may be related to aging.

In addition, we found that pyruvic acid (the final product of the glycolytic pathway) in PABS showed a significant downward trend compared with HCs and ABS. The human brain is a highly metabolized organ, and its energy requirements are almost entirely derived from the metabolism of glucose and pyruvic acid [54]. In our research, we found that pyruvic acid metabolism is still disordered after several months of heroin withdrawal, suggesting that heroin may have chronic and lasting damage to the central nervous system. Pyruvic acid can be converted into lactic acid in the cytoplasm to provide energy, and it can be oxidized and decarboxylated into acetyl-CoA in the mitochondria; from there, it enters the TCA cycle to be oxidized to carbon dioxide and water [55]. Interestingly, the decrease of pyruvate in PABS did not prevent its upregulation in TCA cycle, and the lactic acid level in ABS was also significantly lower than it was in HCs and PABS. Recently, lactic acid has been considered a new type of signaling molecule involved in many key reactions, and it plays an indispensable role in learning and memory, which may be related to drug addiction [56, 57]. Lactate at physiological concentrations functions as a signaling molecule instead of an energy substrate [57], and studies have shown that peripheral administration of lactic acid produces antidepressant-like effects in different animal models of depression [58]. This finding suggests that the low lactate level may be closely related to acute withdrawal.

### Effects of heroin on metabolites persist after prolonged withdrawal

Heroin has a lasting effect on the metabolism of the human body. Even after several months of heroin withdrawal, four metabolites (alpha-aminobutyric acid, alloisoleucine, ketoleucine, and oxalic acid) remain unrecovered. Alpha-aminobutyric acid is closely related to the metabolism of glutathione in the body [59]. Glutathione deficiency leads to oxidative stress, which plays a key role in the pathogenesis of aging and many diseases [60]. Under oxidative stress, the content of alpha-aminobutyric acid in cells will decrease, which suggests that alpha-aminobutyric acid may be related to aging or other diseases [59, 61]. For example, alpha-aminobutyric acid is closely related to depression symptoms in the elderly [62]. After heroin withdrawal, alpha-aminobutyric acid stays at low levels for a long time, which may be the result of heroin’s oxidative damage to cells [63, 64]. Alloisoleucine is a stereoisomer of isoleucine; ketoleucine is produced by the incomplete breakdown of branched chain AAs. Both of them are related to the metabolism of branched chain AAs, and the reduction of branched chain AAs in the blood is one of the most consistent characteristics of aging [61]. Branched chain AAs can regulate cell senescence through the mechanical target of rapamycin (mTOR). Such as autophagy, cell growth, apoptosis, cell senescence, stem cell and mitochondrial function [65]. In addition, animal experiments showed that supplementation of branched chain amino acids has an important anti-aging effect [66]. Our data indicate that heroin may accelerate the aging of the human body, and this effect still exists even after long-term heroin withdrawal. Oxalic acid is produced by the decomposition of ascorbic acid, which is a powerful antioxidant that can scavenge free radicals in different tissues including the central nervous system [67]. The decrease of oxalic acid may indicate that the metabolism of ascorbic acid is affected, which aggravates oxidative stress in the body and promotes inflamm-aging [68]. In addition, recent studies have shown that gut microbes play an important role in the metabolism of oxalic acid [69, 70]. Many microbiota, such as Oxalobacter formigenes, Lactobacilli, and Bifidobacteria, are involved in the degradation of oxalic acid. According to our previous research [71], substance use disorders (including heroin abuse) can significantly change the gut microbiota. The persistent low level of oxalic acid during the heroin withdrawal may be related to and imbalance of gut microbes.

## CONCLUSIONS

Compared with other diseases, human metabolomics data in the field of drug addiction remain limited. Our data support the findings from previous animal studies and we found that metabolism of heroin addicts in the acute withdrawal period is significantly different from that in the late withdrawal period. In particular, the metabolism of essential FAs, neurotransmitter precursors and energy production pathways were obviously abnormal, and these insights might provide a strategy for intervention in heroin addicts during withdrawal.

## MATERIALS AND METHODS

### Patients

In this case-control study, a total of 35 men with heroin withdrawal were recruited from a joint program of drug detoxification and rehabilitation in the First Affiliated Hospital of Kunming Medical University and the Kunming Drug Rehabilitation Center from January 2018 to October 2019, including 20 patients with acute abstinence stage (ABS) and 15 patients with protracted abstinence stage (PABS). All patients had a clear history of heroin use that was confirmed by urine screening tests. Fifteen age-, sex-, and body mass index–matched non-heroin-use volunteers were enrolled as healthy controls (HCs). All protocols and recruitment procedures described in this study were approved by the Research Ethics Committee of the First Affiliated Hospital of Kunming Medical University (2018-L-42), and the study was conducted according to the tenets of the Declaration of Helsinki. All participants provided written informed consent before enrollment.

### Sample preparation, derivatization, and UPLC-MS/MS analysis

Blood samples were collected and stored in the First Affiliated Hospital of Kunming Medical University Biobank using standard procedures. Peripheral venous blood was collected from fasted participants with heroin withdrawal and from HCs between 08:00 and 10:00 AM using Vacutainer blood collection tubes with EDTA as an anticoagulant. The anticoagulant-treated blood samples were gently mixed by inverting the tube several times. Blood samples were then centrifuged at 3,000 g for 10 min at 4° C. The upper layer, containing plasma, was transferred to a 2-mL Eppendorf tube (500 μL per tube) and immediately stored at −80° C until use. The protocols for preparation and derivatization of plasma samples were based on a previously published method, with minor modifications. In brief, 50 μL of plasma was weighed and transferred to a new 1.5-mL tube, and 20 μL of methanol/chloroform/water (3:1:1) was added. The sample was homogenized with zirconium oxide beads for 3 min and then was centrifuged at 18,000 g for 20 min. The supernatant was transferred to a 96-well plate, and 20 μL of freshly prepared derivative reagents was added to each well. The plate was sealed, and the derivatization was carried out at 30° C for 60 min. After derivatization, 350 μL of supernatant was transferred to a new 96-well plate with 15 μL internal standards in each well. Serial dilutions of derivatized stock standards were added to the left well. Finally, the plate was sealed for UPLC-MS/MS analysis.

### Multivariate statistical analysis

Demographic, continuous variables were expressed as the mean ± standard deviation. We used the Wilcoxon test to analyze continuous variables and either the Fisher test or chi squared test to analyze categorical variables. A p value < 0.05 was considered statistically significant. The raw data files generated by UPLC-MS/MS were processed using the iMAP platform (v1.0; Metabo-Profile, Shanghai, China). Partial least squares-discriminant analysis (PLS-DA) and orthogonal partial least squares discriminant analysis (OPLS-DA) were also performed. PCA is an unsupervised data analysis. It can be used to evaluate whether possible abnormal samples and outliers were present through the aggregation/separation of observation points. Each point represents a sample. If points exceed the 95% confidence interval, they will appear outside the ellipse and may be an outlier sample. PC1 is the first principal component of the model, and PC2 is the second principal component of the model. OPLS-DA was used to distinguish the differences in metabolic profiles between the two groups. In the OPLS-DA score plot, the abscissa P1 represents the first predicted principal component of the model, the ordinate O1 represents the first orthogonal component of the model, and the percentage in parentheses represents the interpretation rate of the principal component. The variable importance in projection (VIP) was obtained according to the OPLS-DA model. Metabolites with VIPs of ≥ 1 and p < 0.05 were regarded as statistically significant. (Univariate analyses were applied when the data were normally distributed.) The heat plot of metabolites was formed with the iMAP platform, after unit variance scaling for each metabolite. To explore the related metabolic pathway disruptions in more detail, iMAP platform, Human Metabolome Database, and the Kyoto Encyclopedia of Genes and Genomes were used.

## Supplementary Material

Supplementary Figure 1

Supplementary Table 1

Supplementary Table 2

## References

[1] Kolodny A, Courtwright DT, Hwang CS, Kreiner P, Eadie JL, Clark TW, Alexander GC. The prescription opioid and heroin crisis: a public health approach to an epidemic of addiction. Annu Rev Public Health. 2015; 36:559–74. 10.1146/annurev-publhealth-031914-122957 25581144

[2] Csete J, Kamarulzaman A, Kazatchkine M, Altice F, Balicki M, Buxton J, Cepeda J, Comfort M, Goosby E, Goulão J, Hart C, Kerr T, Lajous AM, et al. Public health and international drug policy. Lancet. 2016; 387:1427–80. 10.1016/S0140-6736(16)00619-X 27021149 PMC5042332

[3] Goldstein RZ, Volkow ND. Drug addiction and its underlying neurobiological basis: neuroimaging evidence for the involvement of the frontal cortex. Am J Psychiatry. 2002; 159:1642–52. 10.1176/appi.ajp.159.10.1642 12359667 PMC1201373

[4] Hosztafi S. [Heroin addiction]. Acta Pharm Hung. 2011; 81:173–83. 22329304

[5] Clinical Guidelines for Withdrawal Management and Treatment of Drug Dependence in Closed Settings. Geneva: World Health Organization. 2009. 26269862

[6] Robbins TW, Ersche KD, Everitt BJ. Drug addiction and the memory systems of the brain. Ann N Y Acad Sci. 2008; 1141:1–21. 10.1196/annals.1441.020 18991949

[7] Gould TJ. Addiction and cognition. Addict Sci Clin Pract. 2010; 5:4–14. 22002448 PMC3120118

[8] Zou F, Wu X, Zhai T, Lei Y, Shao Y, Jin X, Tan S, Wu B, Wang L, Yang Z. Abnormal resting-state functional connectivity of the nucleus accumbens in multi-year abstinent heroin addicts. J Neurosci Res. 2015; 93:1693–702. 10.1002/jnr.23608 26280556

[9] Wang L, Zou F, Zhai T, Lei Y, Tan S, Jin X, Ye E, Shao Y, Yang Y, Yang Z. Abnormal gray matter volume and resting-state functional connectivity in former heroin-dependent individuals abstinent for multiple years. Addict Biol. 2016; 21:646–56. 10.1111/adb.12228 25727574

[10] Dole VP. Narcotic addiction, physical dependence and relapse. N Engl J Med. 1972; 286:988–92. 10.1056/NEJM197205042861808 4622638

[11] Shima N, Miyawaki I, Bando K, Horie H, Zaitsu K, Katagi M, Bamba T, Tsuchihashi H, Fukusaki E. Influences of methamphetamine-induced acute intoxication on urinary and plasma metabolic profiles in the rat. Toxicology. 2011; 287:29–37. 10.1016/j.tox.2011.05.012 21645582

[12] Kaplan KA, Chiu VM, Lukus PA, Zhang X, Siems WF, Schenk JO, Hill HH Jr. Neuronal metabolomics by ion mobility mass spectrometry: cocaine effects on glucose and selected biogenic amine metabolites in the frontal cortex, striatum, and thalamus of the rat. Anal Bioanal Chem. 2013; 405:1959–68. 10.1007/s00216-012-6638-7 23314481

[13] Zaitsu K, Miyawaki I, Bando K, Horie H, Shima N, Katagi M, Tatsuno M, Bamba T, Sato T, Ishii A, Tsuchihashi H, Suzuki K, Fukusaki E. Metabolic profiling of urine and blood plasma in rat models of drug addiction on the basis of morphine, methamphetamine, and cocaine-induced conditioned place preference. Anal Bioanal Chem. 2014; 406:1339–54. 10.1007/s00216-013-7234-1 23912828

[14] Picca A, Calvani R, Landi G, Marini F, Biancolillo A, Gervasoni J, Persichilli S, Primiano A, Urbani A, Bossola M, Bentivoglio AR, Cesari M, Landi F, et al. Circulating amino acid signature in older people with Parkinson’s disease: A metabolic complement to the EXosomes in PArkiNson Disease (EXPAND) study. Exp Gerontol. 2019; 128:110766. 10.1016/j.exger.2019.110766 31666195

[15] Socha E, Koba M, Kośliński P. Amino acid profiling as a method of discovering biomarkers for diagnosis of neurodegenerative diseases. Amino Acids. 2019; 51:367–71. 10.1007/s00726-019-02705-6 30725224

[16] Beasley CL, Honer WG, Ramos-Miguel A, Vila-Rodriguez F, Barr AM. Prefrontal fatty acid composition in schizophrenia and bipolar disorder: Association with reelin expression. Schizophr Res. 2020; 215:493–98. 10.1016/j.schres.2017.05.033 28583708

[17] Hosseini M, Poljak A, Braidy N, Crawford J, Sachdev P. Blood fatty acids in Alzheimer’s disease and mild cognitive impairment: A meta-analysis and systematic review. Ageing Res Rev. 2020; 60:101043. 10.1016/j.arr.2020.101043 32194194

[18] Youdim KA, Martin A, Joseph JA. Essential fatty acids and the brain: possible health implications. Int J Dev Neurosci. 2000; 18:383–99. 10.1016/s0736-5748(00)00013-7 10817922

[19] Bazinet RP, Layé S. Polyunsaturated fatty acids and their metabolites in brain function and disease. Nat Rev Neurosci. 2014; 15:771–85. 10.1038/nrn3820 25387473

[20] Nasaruddin ML, Pan X, McGuinness B, Passmore P, Kehoe PG, Hölscher C, Graham SF, Green BD. Evidence That Parietal Lobe Fatty Acids May Be More Profoundly Affected in Moderate Alzheimer’s Disease (AD) Pathology Than in Severe AD Pathology. Metabolites. 2018; 8:69. 10.3390/metabo8040069 30373213 PMC6316131

[21] Messamore E, Almeida DM, Jandacek RJ, McNamara RK. Polyunsaturated fatty acids and recurrent mood disorders: Phenomenology, mechanisms, and clinical application. Prog Lipid Res. 2017; 66:1–13. 10.1016/j.plipres.2017.01.001 28069365 PMC5422125

[22] Smith AM, King JJ, West PR, Ludwig MA, Donley EL, Burrier RE, Amaral DG. Amino Acid Dysregulation Metabotypes: Potential Biomarkers for Diagnosis and Individualized Treatment for Subtypes of Autism Spectrum Disorder. Biol Psychiatry. 2019; 85:345–54. 10.1016/j.biopsych.2018.08.016 30446206 PMC6837735

[23] Benson KL, Bottary R, Schoerning L, Baer L, Gonenc A, Eric Jensen J, Winkelman JW. ^1^H MRS Measurement of Cortical GABA and Glutamate in Primary Insomnia and Major Depressive Disorder: Relationship to Sleep Quality and Depression Severity. J Affect Disord. 2020; 274:624–31. 10.1016/j.jad.2020.05.026 32663996 PMC10662933

[24] Yu H, Hong S, Jeong CH, Bae JW, Lee S. Development of a linear dual column HPLC-MS/MS method and clinical genetic evaluation for tramadol and its phase I and II metabolites in oral fluid. Arch Pharm Res. 2018; 41:288–98. 10.1007/s12272-017-0993-z 29196917

[25] Naveed M, Mubeen S, Khan A, Ibrahim S, Meer B. Plasma Biomarkers: Potent Screeners of Alzheimer’s Disease. Am J Alzheimers Dis Other Demen. 2019; 34:290–301. 10.1177/1533317519848239 31072117 PMC10852434

[26] Yang J, Chen T, Sun L, Zhao Z, Qi X, Zhou K, Cao Y, Wang X, Qiu Y, Su M, Zhao A, Wang P, Yang P, et al. Potential metabolite markers of schizophrenia. Mol Psychiatry. 2013; 18:67–78. 10.1038/mp.2011.131 22024767 PMC3526727

[27] Davison J, O’Gorman A, Brennan L, Cotter DR. A systematic review of metabolite biomarkers of schizophrenia. Schizophr Res. 2018; 195:32–50. 10.1016/j.schres.2017.09.021 28947341

[28] Hibbeln JR, Salem N Jr. Dietary polyunsaturated fatty acids and depression: when cholesterol does not satisfy. Am J Clin Nutr. 1995; 62:1–9. 10.1093/ajcn/62.1.1 7598049

[29] Innes JK, Calder PC. Omega-6 fatty acids and inflammation. Prostaglandins Leukot Essent Fatty Acids. 2018; 132:41–48. 10.1016/j.plefa.2018.03.004 29610056

[30] Xie P, Wang TJ, Yin G, Yan Y, Xiao LH, Li Q, Bi KS. Metabonomic Study of Biochemical Changes in Human Hair of Heroin Abusers by Liquid Chromatography Coupled with Ion Trap-Time of Flight Mass Spectrometry. J Mol Neurosci. 2016; 58:93–101. 10.1007/s12031-015-0655-x 26445826

[31] Thies F, Miles EA, Nebe-von-Caron G, Powell JR, Hurst TL, Newsholme EA, Calder PC. Influence of dietary supplementation with long-chain n-3 or n-6 polyunsaturated fatty acids on blood inflammatory cell populations and functions and on plasma soluble adhesion molecules in healthy adults. Lipids. 2001; 36:1183–93. 10.1007/s11745-001-0831-4 11795850

[32] Miles EA, Allen E, Calder PC. *In vitro* effects of eicosanoids derived from different 20-carbon Fatty acids on production of monocyte-derived cytokines in human whole blood cultures. Cytokine. 2002; 20:215–23. 10.1006/cyto.2002.2007 12550106

[33] Kakutani S, Ishikura Y, Tateishi N, Horikawa C, Tokuda H, Kontani M, Kawashima H, Sakakibara Y, Kiso Y, Shibata H, Morita I. Supplementation of arachidonic acid-enriched oil increases arachidonic acid contents in plasma phospholipids, but does not increase their metabolites and clinical parameters in Japanese healthy elderly individuals: a randomized controlled study. Lipids Health Dis. 2011; 10:241. 10.1186/1476-511X-10-241 22188761 PMC3314585

[34] Chevalier G, Siopi E, Guenin-Macé L, Pascal M, Laval T, Rifflet A, Boneca IG, Demangel C, Colsch B, Pruvost A, Chu-Van E, Messager A, Leulier F, et al. Effect of gut microbiota on depressive-like behaviors in mice is mediated by the endocannabinoid system. Nat Commun. 2020; 11:6363. 10.1038/s41467-020-19931-2 33311466 PMC7732982

[35] LopezLB, Kritz-SilversteinD, Barrett ConnorE. High dietary and plasma levels of the omega-3 fatty acid docosahexaenoic acid are associated with decreased dementia risk: the Rancho Bernardo study.J Nutr Health Aging. 2011; 15:25–31. 10.1007/s12603-011-0009-521267518

[36] Shahidi F, Ambigaipalan P. Omega-3 Polyunsaturated Fatty Acids and Their Health Benefits. Annu Rev Food Sci Technol. 2018; 9:345–81. 10.1146/annurev-food-111317-095850 29350557

[37] Thesing CS, Bot M, Milaneschi Y, Giltay EJ, Penninx BW. Omega-3 and omega-6 fatty acid levels in depressive and anxiety disorders. Psychoneuroendocrinology. 2018; 87:53–62. 10.1016/j.psyneuen.2017.10.005 29040890

[38] Gutiérrez S, Svahn SL, Johansson ME. Effects of Omega-3 Fatty Acids on Immune Cells. Int J Mol Sci. 2019; 20:5028. 10.3390/ijms20205028 31614433 PMC6834330

[39] Grosso G, Galvano F, Marventano S, Malaguarnera M, Bucolo C, Drago F, Caraci F. Omega-3 fatty acids and depression: scientific evidence and biological mechanisms. Oxid Med Cell Longev. 2014; 2014:313570. 10.1155/2014/313570 24757497 PMC3976923

[40] Zhang YP, Miao R, Li Q, Wu T, Ma F. Effects of DHA Supplementation on Hippocampal Volume and Cognitive Function in Older Adults with Mild Cognitive Impairment: A 12-Month Randomized, Double-Blind, Placebo-Controlled Trial. J Alzheimers Dis. 2017; 55:497–507. 10.3233/JAD-160439 27716665

[41] Leshner AI. Addiction is a brain disease, and it matters. Science. 1997; 278:45–47. 10.1126/science.278.5335.45 9311924

[42] Tomkins DM, Sellers EM. Addiction and the brain: the role of neurotransmitters in the cause and treatment of drug dependence. CMAJ. 2001; 164:817–21. 11276551 PMC80880

[43] Capuron L, Schroecksnadel S, Féart C, Aubert A, Higueret D, Barberger-Gateau P, Layé S, Fuchs D. Chronic low-grade inflammation in elderly persons is associated with altered tryptophan and tyrosine metabolism: role in neuropsychiatric symptoms. Biol Psychiatry. 2011; 70:175–82. 10.1016/j.biopsych.2010.12.006 21277567

[44] Hüfner K, Fuchs D, Blauth M, Sperner-Unterweger B. How acute and chronic physical disease may influence mental health - An Analysis of neurotransmitter precursor amino acid levels. Psychoneuroendocrinology. 2019; 106:95–101. 10.1016/j.psyneuen.2019.03.028 30959235

[45] Comai S, Bertazzo A, Brughera M, Crotti S. Tryptophan in health and disease. Adv Clin Chem. 2020; 95:165–218. 10.1016/bs.acc.2019.08.005 32122523

[46] Gilmour DG, Manowitz P, Frosch WA, Shopsin B. Association of plasma tryptophan levels with clinical change in female schizophrenic patients. Biol Psychiatry. 1973; 6:119–28. 4709129

[47] Molina JA, Jiménez-Jiménez FJ, Gomez P, Vargas C, Navarro JA, Ortí-Pareja M, Gasalla T, Benito-León J, Bermejo F, Arenas J. Decreased cerebrospinal fluid levels of neutral and basic amino acids in patients with Parkinson’s disease. J Neurol Sci. 1997; 150:123–27. 10.1016/s0022-510x(97)00069-5 9268238

[48] Fekkes D, van der Cammen TJ, van Loon CP, Verschoor C, van Harskamp F, de Koning I, Schudel WJ, Pepplinkhuizen L. Abnormal amino acid metabolism in patients with early stage Alzheimer dementia. J Neural Transm (Vienna). 1998; 105:287–94. 10.1007/s007020050058 9660107

[49] Ogawa S, Fujii T, Koga N, Hori H, Teraishi T, Hattori K, Noda T, Higuchi T, Motohashi N, Kunugi H. Plasma L-tryptophan concentration in major depressive disorder: new data and meta-analysis. J Clin Psychiatry. 2014; 75:e906–15. 10.4088/JCP.13r08908 25295433

[50] Chiappelli J, Postolache TT, Kochunov P, Rowland LM, Wijtenburg SA, Shukla DK, Tagamets M, Du X, Savransky A, Lowry CA, Can A, Fuchs D, Hong LE. Tryptophan Metabolism and White Matter Integrity in Schizophrenia. Neuropsychopharmacology. 2016; 41:2587–95. 10.1038/npp.2016.66 27143602 PMC4987857

[51] Zheng T, Liu L, Aa J, Wang G, Cao B, Li M, Shi J, Wang X, Zhao C, Gu R, Zhou J, Xiao W, Yu X, et al. Metabolic phenotype of rats exposed to heroin and potential markers of heroin abuse. Drug Alcohol Depend. 2013; 127:177–86. 10.1016/j.drugalcdep.2012.06.031 22840430

[52] Asadi Shahmirzadi A, Edgar D, Liao CY, Hsu YM, Lucanic M, Asadi Shahmirzadi A, Wiley CD, Gan G, Kim DE, Kasler HG, Kuehnemann C, Kaplowitz B, Bhaumik D, et al. Alpha-Ketoglutarate, an Endogenous Metabolite, Extends Lifespan and Compresses Morbidity in Aging Mice. Cell Metab. 2020; 32:447–456.e6. 10.1016/j.cmet.2020.08.004 32877690 PMC8508957

[53] Sharma R, Ramanathan A. The Aging Metabolome-Biomarkers to Hub Metabolites. Proteomics. 2020; 20:e1800407. 10.1002/pmic.201800407 32068959 PMC7117067

[54] Amaral AI. Effects of hypoglycaemia on neuronal metabolism in the adult brain: role of alternative substrates to glucose. J Inherit Metab Dis. 2013; 36:621–34. 10.1007/s10545-012-9553-3 23109064

[55] Gray LR, Tompkins SC, Taylor EB. Regulation of pyruvate metabolism and human disease. Cell Mol Life Sci. 2014; 71:2577–604. 10.1007/s00018-013-1539-2 24363178 PMC4059968

[56] Wang J, Cui Y, Yu Z, Wang W, Cheng X, Ji W, Guo S, Zhou Q, Wu N, Chen Y, Chen Y, Song X, Jiang H, et al. Brain Endothelial Cells Maintain Lactate Homeostasis and Control Adult Hippocampal Neurogenesis. Cell Stem Cell. 2019; 25:754–767.e9. 10.1016/j.stem.2019.09.009 31761722

[57] Wang Q, Hu Y, Wan J, Dong B, Sun J. Lactate: A Novel Signaling Molecule in Synaptic Plasticity and Drug Addiction. Bioessays. 2019; 41:e1900008. 10.1002/bies.201900008 31270822

[58] Carrard A, Elsayed M, Margineanu M, Boury-Jamot B, Fragnière L, Meylan EM, Petit JM, Fiumelli H, Magistretti PJ, Martin JL. Peripheral administration of lactate produces antidepressant-like effects. Mol Psychiatry. 2018; 23:392–99. 10.1038/mp.2016.179 27752076 PMC5794893

[59] Irino Y, Toh R, Nagao M, Mori T, Honjo T, Shinohara M, Tsuda S, Nakajima H, Satomi-Kobayashi S, Shinke T, Tanaka H, Ishida T, Miyata O, Hirata KI. 2-Aminobutyric acid modulates glutathione homeostasis in the myocardium. Sci Rep. 2016; 6:36749. 10.1038/srep36749 27827456 PMC5101505

[60] Wu G, Fang YZ, Yang S, Lupton JR, Turner ND. Glutathione metabolism and its implications for health. J Nutr. 2004; 134:489–92. 10.1093/jn/134.3.489 14988435

[61] Le Couteur DG, Ribeiro R, Senior A, Hsu B, Hirani V, Blyth FM, Waite LM, Simpson SJ, Naganathan V, Cumming RG, Handelsman DJ. Branched Chain Amino Acids, Cardiometabolic Risk Factors and Outcomes in Older Men: The Concord Health and Ageing in Men Project. J Gerontol A Biol Sci Med Sci. 2020; 75:1805–10. 10.1093/gerona/glz192 31428789

[62] Adachi Y, Toyoshima K, Nishimoto R, Ueno S, Tanaka T, Imaizumi A, Arashida N, Nakamura M, Abe Y, Hakamada T, Kaneko E, Takahashi S, Jinzu H, Shimokado K. Association between plasma α-aminobutyric acid and depressive symptoms in older community-dwelling adults in Japan. Geriatr Gerontol Int. 2019; 19:254–58. 10.1111/ggi.13585 30561103

[63] Xu B, Wang Z, Li G, Li B, Lin H, Zheng R, Zheng Q. Heroin-administered mice involved in oxidative stress and exogenous antioxidant-alleviated withdrawal syndrome. Basic Clin Pharmacol Toxicol. 2006; 99:153–61. 10.1111/j.1742-7843.2006.pto_461.x 16918717

[64] Bani-Ahmad MA, Mustafa AG, Ahmad A, Rahim A. Assessment of oxidative stress of platelets among chronic heroin and hashish addicts. Hum Exp Toxicol. 2018; 37:1017–24. 10.1177/0960327118756721 29405767

[65] Liu GY, Sabatini DM. mTOR at the nexus of nutrition, growth, ageing and disease. Nat Rev Mol Cell Biol. 2020; 21:183–203. 10.1038/s41580-019-0199-y 31937935 PMC7102936

[66] D’Antona G, Ragni M, Cardile A, Tedesco L, Dossena M, Bruttini F, Caliaro F, Corsetti G, Bottinelli R, Carruba MO, Valerio A, Nisoli E. Branched-chain amino acid supplementation promotes survival and supports cardiac and skeletal muscle mitochondrial biogenesis in middle-aged mice. Cell Metab. 2010; 12:362–72. 10.1016/j.cmet.2010.08.016 20889128

[67] Monacelli F, Acquarone E, Giannotti C, Borghi R, Nencioni A. Vitamin C, Aging and Alzheimer’s Disease. Nutrients. 2017; 9:670. 10.3390/nu9070670 28654021 PMC5537785

[68] Knight J, Madduma-Liyanage K, Mobley JA, Assimos DG, Holmes RP. Ascorbic acid intake and oxalate synthesis. Urolithiasis. 2016; 44:289–97. 10.1007/s00240-016-0868-7 27002809 PMC4946963

[69] Crivelli JJ, Mitchell T, Knight J, Wood KD, Assimos DG, Holmes RP, Fargue S. Contribution of Dietary Oxalate and Oxalate Precursors to Urinary Oxalate Excretion. Nutrients. 2020; 13:62. 10.3390/nu13010062 33379176 PMC7823532

[70] Ticinesi A, Nouvenne A, Chiussi G, Castaldo G, Guerra A, Meschi T. Calcium Oxalate Nephrolithiasis and Gut Microbiota: Not just a Gut-Kidney Axis. A Nutritional Perspective. Nutrients. 2020; 12:548. 10.3390/nu12020548 32093202 PMC7071363

[71] Xu Y, Xie Z, Wang H, Shen Z, Guo Y, Gao Y, Chen X, Wu Q, Li X, Wang K. Bacterial Diversity of Intestinal Microbiota in Patients with Substance Use Disorders Revealed by 16S rRNA Gene Deep Sequencing. Sci Rep. 2017; 7:3628. 10.1038/s41598-017-03706-9 28620208 PMC5472629

