# Supplementary material for: Plasma metabolites changes in male heroin addicts during acute and protracted withdrawal
Source: Aging (Albany NY). 2021 Jul 19;13(14):18669–88. doi: 10.18632/aging.203311 (PMC8351709; doi:10.18632/aging.203311)
Supplement: Supplementary Figure 1 [file aging-13-203311-s001.pdf]

SUPPLEMENTARY FIGURE

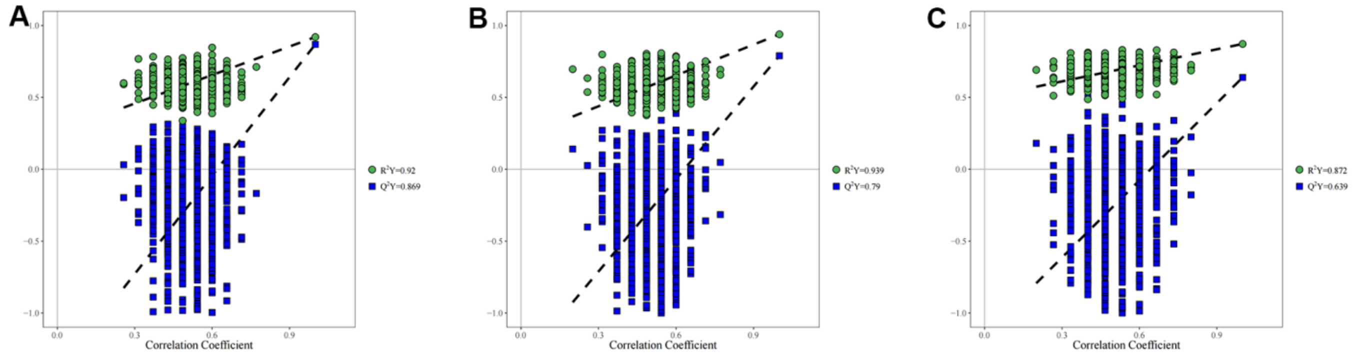

Supplementary Figure 1. Validation plot the OPLS-DA obtained from 1,000-times permutation tests. (**A**) Statistical validation of the OPLS-DA model by permutation testing between healthy controls and acute heroin withdrawal. (**B**) Statistical validation of the OPLS-DA model by permutation testing between long-term heroin withdrawal and acute heroin withdrawal. (**C**) Statistical validation of the OPLS-DA model by permutation testing between healthy controls and long-term heroin withdrawal.
