# Supplementary material for: Plasma metabolites changes in male heroin addicts during acute and protracted withdrawal
Source: Aging (Albany NY). 2021 Jul 19;13(14):18669–88. doi: 10.18632/aging.203311 (PMC8351709; doi:10.18632/aging.203311)
Supplement: Supplementary Table 1 [file aging-13-203311-s002.docx]

**Supplementary Table 1. 129 plasma metabolites identified as endogenous metabolites by UPLC-MS/MS analysis.**

| **Class** | **Metabolite** | **ABS** | **PABS** | **HCs** | **FDR** |
| --- | --- | --- | --- | --- | --- |
| Amino acids | Lysine | 62098 ± 17032 | 71903 ± 15943 | 69506 ± 17946 | 0.274^a^ |
| Amino acids | Histidine | 30721 ± 7294 | 46027 ± 14052 | 36821 ± 7306 | 0.004^b^ |
| Amino acids | Sarcosine | 953 ± 413 | 1191 ± 453 | 1122 ± 546 | 0.369^a^ |
| Amino acids | beta-Alanine | 1093 ± 302 | 936 ± 294 | 758 ± 328 | 0.019^a^ |
| Amino acids | Alanine | 406551 ± 72447 | 414623 ± 125236 | 447994 ± 86257 | 0.503^a^ |
| Amino acids | Dimethylglycine | 1019 ± 344 | 1246 ± 450 | 783 ± 234 | 0.007^a^ |
| Amino acids | GABA | 290 ± 158 | 294 ± 277 | 398 ± 277 | 0.355^b^ |
| Amino acids | Serine | 189725 ± 5235 | 19020 ± 4302 | 15962 ± 5242 | 0.211^a^ |
| Amino acids | Threonine | 16030 ± 4881 | 16228 ± 2854 | 17983 ± 5640 | 0.552^b^ |
| Amino acids | Homoserine | 769 ± 417 | 1684 ± 1262 | 1338 ± 696 | 0.03^b^ |
| Amino acids | Creatine | 10186 ± 5095 | 10688 ± 3786 | 12474 ± 8022 | 0.593^b^ |
| Amino acids | Ornithine | 401 ± 136 | 445 ± 115 | 411 ± 120 | 0.732^b^ |
| Amino acids | Homocitrulline | 1201 ± 138 | 1066 ± 370 | 1239 ± 845 | 0.35^b^ |
| Amino acids | Methylcysteine | 14925 ± 3923 | 20430 ± 4965 | 16311 ± 15751 | 0.009^b^ |
| Amino acids | 2-Phenylglycine | 475 ± 16 | 494 ± 23 | 476 ± 23 | 0.055^b^ |
| Amino acids | Tyrosine | 113537 ± 25254 | 219155 ± 83218 | 190479 ± 49705 | < 0.001^b^ |
| Amino acids | Asparagine | 295734 ± 66866 | 322539 ± 77632 | 279666 ± 66527 | 0.317^a^ |
| Amino acids | Phenylalanine | 83224 ± 25981 | 122285 ± 26207 | 113721 ± 28328 | 0.001^b^ |
| Amino acids | Aspartic acid | 384455 ± 149561 | 841596 ± 359948 | 718018 ± 298281 | < 0.001^b^ |
| Amino acids | Aminoadipic acid | 2785 ± 1991 | 4669 ± 3067 | 4214 ± 2979 | 0.14^a^ |
| Amino acids | N-Acetylalanine | 3310 ± 300 | 3048 ± 320 | 2948 ± 225 | 0.003^a^ |
| Amino acids | N-Acetylaspartic acid | 433 ± 150 | 577 ± 101 | 485 ± 86 | 0.001^b^ |
| Amino acids | N-Acetyltyrosine | 909 ± 434 | 926 ± 442 | 973 ± 503 | 0.917^a^ |
| Amino acids | Glycine | 39111 ± 16958 | 34688 ± 13062 | 24781 ± 12936 | 0.022^b^ |
| Amino acids | Citrulline | 54137 ± 10108 | 53996 ± 24834 | 51787 ± 17991 | 0.469^b^ |
| Amino acids | alpha-Aminobutyric acid | 2619 ± 1196 | 2997 ± 1004 | 4296 ± 1393 | 0.002^b^ |
| Amino acids | Proline | 325560 ± 87430 | 414660 ± 119356 | 359300 ± 145542 | 0.146^b^ |
| Amino acids | Acetylglycine | 5226 ± 2025 | 5167 ± 1278 | 4658 ± 1124 | 0.552^b^ |
| Amino acids | Pipecolic acid | 9460 ± 7793 | 10002 ± 1939 | 8713 ± 2237 | 0.028^b^ |
| Amino acids | N-Acetylserine | 1847 ± 415 | 2010 ± 256 | 1690 ± 270 | 0.061^a^ |
| Amino acids | N-Acetylglutamine | 735 ± 56 | 746 ± 59 | 732 ± 57 | 0.622^b^ |
| Amino acids | Valine | 151714 ± 27674 | 181578 ± 37367 | 201763 ± 50454 | 0.003^a^ |
| Amino acids | Pyroglutamic acid | 22819 ± 5322 | 43217 ± 12682 | 44936 ± 20275 | < 0.001^b^ |
| Amino acids | 5-Aminolevulinic acid | 207 ± 97 | 235 ± 111 | 212 ± 144 | 0.648^b^ |
| Amino acids | Methionine | 64532 ± 16215 | 87119 ± 24098 | 90761 ± 17276 | 0.002^b^ |
| Amino acids | Alloisoleucine | 16907 ± 10529 | 18001 ± 13386 | 29198 ± 13581 | 0.027^b^ |
| Amino acids | Isoleucine/Leucine | 303929 ± 73375 | 401713 ± 83068 | 395514 ± 83880 | 0.001^b^ |
| Amino acids | Norleucine | 1761 ± 925 | 1739 ± 519 | 2105 ± 555 | 0.055^b^ |
| Amino acids | Tryptophan | 173180 ± 40158 | 271994 ± 67438 | 302930 ± 75830 | < 0.001^b^ |
| Amino acids | Phenylacetylglutamine | 1082 ± 1254 | 920 ± 1312 | 793 ± 1092 | 0.896^b^ |
| Amino acids | Selenomethionine | 1119 ± 131 | 1287 ± 211 | 1215 ± 167 | 0.031^a^ |
| Amino acids | Aminocaproic acid | 180 ± 62 | 184 ± 47 | 193 ± 58 | 0.83^a^ |
| Amino acids | 2-Hydroxyglutaric acid | 1264 ± 148 | 1600 ± 313 | 1513 ± 265 | 0.001^b^ |
| Fatty acids | butyl-2-enoic acid | 244 ± 79 | 296 ± 101 | 203 ± 87 | 0.027^b^ |
| Fatty acids | 2-Hydroxy-3-methylbutyric acid | 17175 ± 9569 | 16141 ± 7442 | 13594 ± 7333 | 0.512^b^ |
| Fatty acids | 2-Methy-4-pentenoic acid | 417 ± 23 | 424 ± 36 | 426 ± 28 | 0.604^b^ |
| Fatty acids | 2-Hydroxycaproic acid | 583 ± 84 | 643 ± 103 | 620 ± 99 | 0.244^a^ |
| Fatty acids | Azelaic acid | 620 ± 45 | 696 ± 38 | 797 ± 409 | < 0.001^b^ |
| Fatty acids | Sebacic acid | 435 ± 62 | 535 ± 74 | 502 ± 69 | 0.001^a^ |
| Fatty acids | Methylsuccinic acid | 1689 ± 71 | 1717 ± 93 | 1656 ± 66 | 0.145^a^ |
| Fatty acids | Adipic acid | 695 ± 186 | 1131 ± 283 | 941 ± 258 | < 0.001^b^ |
| Fatty acids | Methylglutaric acid | 594 ± 92 | 645 ± 77 | 635 ± 78 | 0.224^a^ |
| Fatty acids | Suberic acid | 480 ± 247 | 1027 ± 287 | 997 ± 496 | < 0.001^b^ |
| Fatty acids | 2-Methylhexanoic acid | 1396 ± 880 | 3409 ± 2024 | 4148 ± 4689 | 0.005^b^ |
| Fatty acids | 4-Methylhexanoic acid | 16723 ± 7969 | 44364 ± 33645 | 39295 ± 18386 | < 0.001^b^ |
| Fatty acids | Heptanoic acid | 19532 ± 3368 | 27035 ± 5716 | 23394 ± 5300 | 0.002^b^ |
| Fatty acids | Octanoic acid | 27425 ± 6063 | 35300 ± 9898 | 33260 ± 7417 | 0.022^a^ |
| Fatty acids | Nonanoic acid | 2109 ± 326 | 2994 ± 449 | 2683 ± 513 | < 0.001^a^ |
| Fatty acids | Decanoic acid | 2483 ± 596 | 4851 ± 1687 | 2989 ± 660 | < 0.001^b^ |
| Fatty acids | Undecylenic acid | 1466 ± 471 | 1901 ± 720 | 1494 ± 346 | 0.183^b^ |
| Fatty acids | Undecanoic acid | 976 ± 141 | 1240 ± 135 | 1143 ± 137 | < 0.001^a^ |
| Fatty acids | 5Z-Dodecenoic acid | 1253 ± 545 | 3343 ± 1762 | 1404 ± 522 | < 0.001^b^ |
| Fatty acids | Dodecanoic acid | 3588 ± 1721 | 7731 ± 3252 | 5607 ± 1026 | < 0.001^b^ |
| Fatty acids | 12-Tridecenoic acid | 387 ± 68 | 431 ± 78 | 398 ± 70 | 0.246^a^ |
| Fatty acids | Ricinoleic acid | 11285 ± 8650 | 50226 ± 25053 | 17402 ± 5843 | < 0.001^b^ |
| Fatty acids | Ricinelaidic acid | 12362 ± 21213 | 14880 ± 8149 | 5705 ± 5868 | 0.009^b^ |
| Fatty acids | 12-Hydroxystearic acid | 225229 ± 81086 | 262465 ± 103948 | 358005 ± 180445 | 0.036^b^ |
| Fatty acids | Tridecanoic acid | 2047 ± 378 | 2944 ± 323 | 2674 ± 435 | < 0.001^a^ |
| Fatty acids | Myristoleic acid | 34 ± 77 | 1980 ± 2199 | 243 ± 301 | < 0.001^b^ |
| Fatty acids | 9E-tetradecenoic acid | 65 ± 191 | 3565 ± 3838 | 528 ± 588 | < 0.001^b^ |
| Fatty acids | Myristic acid | 9200 ± 3524 | 35167 ± 18754 | 18168 ± 6737 | < 0.001^b^ |
| Fatty acids | Pentadecanoic acid | 10610 ± 6577 | 28971 ± 12344 | 23446 ± 8835 | < 0.001^b^ |
| Fatty acids | Palmitoleic acid | 8371 ± 6432 | 78621 ± 58646 | 20702 ± 15631 | < 0.001^b^ |
| Fatty acids | 10Z-Heptadecenoic acid | 953 ± 791 | 12371 ± 7779 | 4397 ± 2507 | < 0.001^b^ |
| Fatty acids | Linoleic acid | 107977 ± 57078 | 362081 ± 96731 | 267729 ± 85779 | < 0.001^b^ |
| Fatty acids | Bovinic acid | 10798 ± 5708 | 36208 ± 9674 | 26773 ± 8578 | < 0.001^b^ |
| Fatty acids | Arachidonic acid | 51236 ± 27228 | 185397 ± 90230 | 154038 ± 57888 | < 0.001^b^ |
| Fatty acids | Dihomo-gamma-linolenic acid | 2905 ± 1091 | 12312 ± 5381 | 7259 ± 2671 | < 0.001^b^ |
| Fatty acids | DHA | 17726 ± 7068 | 51514 ± 25914 | 51728 ± 20030 | < 0.001^b^ |
| Fatty acids | DPA | 888 ± 398 | 5715 ± 3200 | 3730 ± 1484 | < 0.001^b^ |
| Fatty acids | n-6–DPA | 902 ± 384 | 1566 ± 457 | 1397 ± 264 | < 0.001^b^ |
| Fatty acids | Palmitic acid | 366345 ± 102476 | 733413 ± 150597 | 608533 ± 144258 | < 0.001^b^ |
| Fatty acids | Heptadecanoic acid | 22932 ± 5369 | 56955 ± 12475 | 49094 ± 7791 | < 0.001^b^ |
| Fatty acids | Oleic acid | 347138 ± 223118 | 1494889 ± 441582 | 1090615 ± 445196 | < 0.001^b^ |
| Fatty acids | Stearic acid | 3316303 ± 943153 | 9697744 ± 2876933 | 8206118 ± 1604201 | < 0.001^b^ |
| Fatty acids | 10Z-Nonadecenoic acid | 1422 ± 608 | 3644 ± 1331 | 2268 ± 423 | < 0.001^b^ |
| Fatty acids | 7-Nonadecenoic acid | 2260 ± 182 | 2781 ± 276 | 2506 ± 110 | < 0.001^b^ |
| Fatty acids | 7-trans-Nonadecenoic acid | 3237 ± 640 | 6016 ± 1424 | 4590 ± 526 | < 0.001^b^ |
| Fatty acids | Eicosadienoic acid | 31882 ± 14179 | 212155 ± 107894 | 105367 ± 49693 | < 0.001^b^ |
| Fatty acids | 11-cis-Eicosenoic acid | 10976 ± 4190 | 87070 ± 51306 | 48847 ± 17481 | < 0.001^b^ |
| Fatty acids | 5-Eicosenoic acid | 21044 ± 4217 | 30422 ± 16200 | 24010 ± 5318 | 0.125^b^ |
| Fatty acids | 12,15-Heneicosadienoic acid | 3005 ± 194 | 3046 ± 433 | 2995 ± 226 | 0.736^b^ |
| Fatty acids | 13,16,19-Docosatrienoic acid | 1726 ± 741 | 3439 ± 1469 | 2578 ± 657 | < 0.001^b^ |
| Fatty acids | Citramalic acid | 79 ± 41 | 69 ± 15 | 87 ± 38 | 0.573^b^ |
| Organic acids | Guanidoacetic acid | 878 ± 498 | 969 ± 509 | 1005 ± 556 | 0.848^b^ |
| Organic acids | Hydroxypropionic acid | 44835 ± 7503 | 44617 ± 4431 | 45833 ± 4345 | 0.891^b^ |
| Organic acids | Lactic acid | 1048977 ± 240783 | 1480185 ± 248874 | 1428307 ± 232911 | < 0.001^a^ |
| Organic acids | Malic acid | 3589 ± 793 | 4648 ± 1254 | 4428 ± 1276 | 0.027^a^ |
| Organic acids | Methylmalonic acid | 3975 ± 657 | 5184 ± 2174 | 4429 ± 516 | 0.091^b^ |
| Organic acids | Fumaric acid | 819 ± 204 | 970 ± 256 | 1053 ± 411 | 0.214^b^ |
| Organic acids | Glutaric acid | 726 ± 153 | 904 ± 163 | 828 ± 92 | 0.005^a^ |
| Organic acids | trans-Aconitic acid | 69205 ± 16514 | 79419 ± 19054 | 72880 ± 23803 | 0.348^b^ |
| Organic acids | alpha-Ketoisovaleric acid | 39672 ± 9038 | 49914 ± 10530 | 55958 ± 15433 | 0.002^a^ |
| Organic acids | Ketoleucine | 230250 ± 74987 | 268048 ± 81500 | 374202 ± 112143 | 0.001^b^ |
| Organic acids | 3-Methy-2-oxovaleric acid | 53219 ± 15723 | 56954 ± 14807 | 76096 ± 23681 | 0.004^a^ |
| Organic acids | Glycolic acid | 9898 ± 1160 | 12420 ± 3233 | 10410 ± 2911 | 0.073^b^ |
| Organic acids | 3-Hydroxybutyric acid | 85878 ± 109079 | 200953 ± 166607 | 106079 ± 123629 | 0.028^b^ |
| Organic acids | alpha-Hydroxyisobutyric acid | 15437 ± 2839 | 15046 ± 2242 | 15901 ± 2141 | 0.689^a^ |
| Organic acids | 2-Hydroxybutyric acid | 26302 ± 17261 | 31064 ± 11805 | 37795 ± 23217 | 0.091^b^ |
| Organic acids | Glutaconic acid | 1730 ± 425 | 2047 ± 760 | 1764 ± 666 | 0.365^b^ |
| Organic acids | Malonic acid | 330 ± 62 | 296 ± 44 | 337 ± 96 | 0.369^b^ |
| Organic acids | Succinic acid | 3354 ± 444 | 4215 ± 1515 | 3637 ± 365 | 0.049^b^ |
| Organic acids | Oxalic acid | 51624 ± 8386 | 75322 ± 14523 | 91458 ± 18575 | < 0.001^b^ |
| Organic acids | Maleic acid | 1482 ± 255 | 1580 ± 472 | 1548 ± 574 | 0.759^b^ |
| Organic acids | cis-Aconitic acid | 67982 ± 17059 | 67938 ± 16678 | 62540 ± 20258 | 0.302^b^ |
| Organic acids | Hydroxypyruvic acid | 5917 ± 3444 | 7115 ± 6592 | 9669 ± 9870 | 0.638^b^ |
| Organic acids | Citric acid | 155670 ± 40382 | 210974 ± 53075 | 200660 ± 72687 | 0.012^b^ |
| Organic acids | Isocitric acid | 6011 ± 1939 | 13868 ± 6007 | 13458 ± 4004 | < 0.001^b^ |
| Organic acids | Pyruvic acid | 1365974 ± 319909 | 681861 ± 202373 | 1317594 ± 527156 | < 0.001^b^ |
| Organic acids | Oxoglutaric acid | 28744 ± 6073 | 57352 ± 22003 | 74828 ± 47228 | < 0.001^b^ |
| Organic acids | Oxoadipic acid | 19 ± 15 | 44 ± 30 | 35 ± 15 | 0.004^b^ |
| SCFAs | Ethylmethylacetic acid | 26377 ± 11581 | 28425 ± 8608 | 30452 ± 15934 | 0.891^b^ |
| SCFAs | Formic acid | 57515 ± 12838 | 68279 ± 10523 | 66953 ± 12339 | 0.033^a^ |
| SCFAs | 3-Hydroxyisovaleric acid | 1235 ± 867 | 1251 ± 361 | 1326 ± 635 | 0.532^b^ |
| SCFAs | Propionic acid | 13067 ± 3327 | 14409 ± 1744 | 13388 ± 3074 | 0.119^b^ |
| SCFAs | Butyric acid | 11185 ± 3659 | 18331 ± 9595 | 16583 ± 9265 | 0.055^b^ |
| SCFAs | Isobutyric acid | 68276 ± 13270 | 89549 ± 14146 | 72724 ± 13993 | < 0.001^a^ |
| SCFAs | Caproic acid | 2832 ± 356 | 4621 ± 991 | 3313 ± 536 | < 0.001^b^ |
| SCFAs | Acetic acid | 234297 ± 63594 | 299756 ± 118486 | 230955 ± 72451 | 0.117^b^ |

Abbreviations: ABS: abstinence syndrome, DHA: docosahexaenoic acid, DPA: docosapentaenoic acid, FDR: false discovery rate, GABA: gamma-aminobutyric acid, HCs: healthy controls, UPLC-MS/MS: ultraperformance liquid chromatography coupled to tandem mass spectrometry, PABS: postabstinence syndrome, SCFAs: short-chain fatty acids.

^a^Analyzed by ANOVA.

^b^Analyzed by the Kruskal-Wallis test.
