# Supplementary material for: Plasma metabolites changes in male heroin addicts during acute and protracted withdrawal
Source: Aging (Albany NY). 2021 Jul 19;13(14):18669–88. doi: 10.18632/aging.203311 (PMC8351709; doi:10.18632/aging.203311)
Supplement: Supplementary Table 2 [file aging-13-203311-s003.pdf]

SUPPLEMENTARY TABLE

Supplementary Table 2. Identified differential metabolites between the ABS, PABS, and HCs.

Metabolites	HCs vs. ABS			HCs vs. PABS			PABS vs. ABS			Class	KEGG
	FC	FDR	VIP	FC	FDR	VIP	FC	FDR	VIP		
10Z-Heptadecenoic acid	0.22	< 0.001	1.65	2.67	0.002	1.88	0.08	< 0.001	1.53	Fatty acids	NA
10Z-Nonadecenoic acid	0.69	< 0.001	1.60	1.40	0.002	1.90	0.49	< 0.001	1.55	Fatty acids	NA
11-cis-Eicosenoic acid	0.22	< 0.001	1.86	1.47	0.009	1.55	0.15	< 0.001	1.56	Fatty acids	C16526
13,16,19-Docosatrienoic acid	0.70	0.003	1.41	NA	NA	NA	0.61	< 0.001	1.38	Fatty acids	NA
2-Hydroxyglutaric acid	0.85	0.003	1.08	NA	NA	NA	0.79	< 0.001	1.11	Amino acids	C01087
2-Methylhexanoic acid	NA	NA	NA	NA	NA	NA	0.48	0.006	1.18	Fatty acids	NA
4-Methylhexanoic acid	0.49	< 0.001	1.33	NA	NA	NA	0.53	< 0.001	1.13	Fatty acids	NA
5Z-Dodecenoic acid	NA	NA	NA	2.23	0.002	1.91	0.42	< 0.001	1.28	Fatty acids	NA
7-Nonadecenoic acid	0.90	< 0.001	1.52	1.07	0.002	1.91	0.84	< 0.001	1.57	Fatty acids	NA
7-trans-Nonadecenoic acid	0.71	< 0.001	1.74	1.24	0.003	1.92	0.58	< 0.001	1.65	Fatty acids	NA
9E-tetradecenoic acid	0.01	0.028	1.20	10.27	0.005	1.56	< 0.001	< 0.001	1.18	Fatty acids	NA
Adipic acid	0.77	0.006	1.01	NA	NA	NA	0.67	< 0.001	1.35	Fatty acids	C06104
Alloisoleucine	0.44	0.015	1.01	NA	NA	NA	NA	NA	NA	Amino acids	NA
alpha-Aminobutyric acid	0.53	0.002	1.17	0.66	0.041	1.37	NA	NA	NA	Amino acids	C02356
alpha-Ketoisovaleric acid	0.64	0.005	1.11	NA	NA	NA	NA	NA	NA	Organic acids	C00141
Arachidonic acid	0.30	< 0.001	1.77	NA	NA	NA	0.24	< 0.001	1.49	Fatty acids	C00219
Aspartic acid	0.48	< 0.001	1.29	NA	NA	NA	0.45	< 0.001	1.22	Amino acids	C00049
Azelaic acid	NA	NA	NA	NA	NA	NA	0.90	< 0.001	1.35	Fatty acids	C08261
beta-Alanine	1.44	0.011	1.13	NA	NA	NA	NA	NA	NA	Amino acids	C00099
Butyl-2-enoic acid	NA	NA	NA	1.35	0.039	1.17	NA	NA	NA	Fatty acids	C01771
Bovinic acid	0.44	< 0.001	1.67	1.41	0.009	1.51	0.31	< 0.001	1.66	Fatty acids	C04056
Butyric acid	NA	NA	NA	NA	NA	NA	0.69	0.027	1.02	SCFAs	C00246
Caproic acid	NA	NA	NA	1.36	0.002	1.80	0.63	< 0.001	1.55	SCFAs	C01585
Decanoic acid	NA	NA	NA	1.67	0.003	1.77	0.52	< 0.001	1.34	Fatty acids	C01571
DHA	0.32	< 0.001	1.72	NA	NA	NA	0.36	< 0.001	1.42	Fatty acids	C06429
Dihomo-gamma-linolenic acid	0.41	< 0.001	1.77	1.50	0.003	1.67	0.28	< 0.001	1.63	Fatty acids	C03242
Dimethylglycine	NA	NA	NA	1.53	0.010	1.48	NA	NA	NA	Amino acids	C01026
Dodecanoic acid	0.64	< 0.001	1.21	NA	NA	NA	0.40	< 0.001	1.26	Fatty acids	C02679
DPA	0.22	< 0.001	1.86	NA	NA	NA	0.17	< 0.001	1.58	Fatty acids	C16513
n-6–DPA	0.76	< 0.001	1.54	NA	NA	NA	0.74	< 0.001	1.38	Fatty acids	NA
Eicosadienoic acid	0.33	< 0.001	1.75	1.92	0.001	1.77	0.17	< 0.001	1.64	Fatty acids	C16525
Glutaric acid	NA	NA	NA	NA	NA	NA	0.80	0.006	1.07	Organic acids	C00489
Heptadecanoic acid	0.47	< 0.001	2.00	NA	NA	NA	0.38	< 0.001	1.77	Fatty acids	NA
Heptanoic acid	NA	NA	NA	NA	NA	NA	0.70	< 0.001	1.23	Fatty acids	C17714
Histidine	NA	NA	NA	NA	NA	NA	0.61	0.002	1.12	Amino acids	C00135
Isobutyric acid	NA	NA	NA	1.23	0.017	1.42	0.76	< 0.001	1.20	SCFAs	C02632
Isocitric acid	0.48	< 0.001	1.57	NA	NA	NA	0.52	< 0.001	1.32	Organic acids	C00311
Isoleucine/leucine	0.81	< 0.001	1.05	NA	NA	NA	NA	NA	NA	Amino acids	NA
Ketoleucine	0.51	< 0.001	1.26	0.72	0.033	1.67	NA	NA	NA	Organic acids	C00233
Lactic acid	0.73	< 0.001	1.26	NA	NA	NA	0.71	< 0.001	1.31	Organic acids	C00186
Linoleic acid	0.44	< 0.001	1.67	1.41	0.009	1.51	0.31	< 0.001	1.66	Fatty acids	C01595
Methionine	0.68	< 0.001	1.37	NA	NA	NA	0.74	0.010	1.03	Amino acids	C00073
Methylcysteine	NA	NA	NA	NA	NA	NA	0.77	0.002	1.10	Amino acids	NA
Myristic acid	0.49	< 0.001	1.55	1.78	0.017	1.64	0.27	< 0.001	1.45	Fatty acids	C06424
Myristoleic acid	0.05	0.034	1.09	12.43	0.005	1.52	0.004	< 0.001	1.15	Fatty acids	C08322
N-Acetylalanine	1.12	< 0.001	1.36	NA	NA	NA	NA	NA	NA	Amino acids	NA
N-Acetylserine	NA	NA	NA	1.19	0.015	1.42	NA	NA	NA	Amino acids	NA
Nonanoic acid	0.79	0.003	1.11	NA	NA	NA	0.70	< 0.001	1.46	Fatty acids	C01601
Oleic acid	0.31	< 0.001	1.72	1.43	0.013	1.43	0.22	< 0.001	1.72	Fatty acids	C00712
Oxalic acid	0.52	< 0.001	1.78	NA	NA	NA	0.69	< 0.001	1.37	Organic acids	C00209

Oxoadipic acid	0.92	0.004	1.15	NA	NA	NA	NA	NA	NA	Organic acids	C00322
Oxoglutaric acid	0.46	< 0.001	1.33	NA	NA	NA	0.55	< 0.001	1.36	Organic acids	C00026
Palmitic acid	0.63	< 0.001	1.65	1.22	0.027	1.35	0.50	< 0.001	1.66	Fatty acids	C00249
Palmitoleic acid	0.38	0.034	1.16	2.64	0.003	1.83	0.14	< 0.001	1.36	Fatty acids	C08362
Pentadecanoic acid	0.45	< 0.001	1.51	NA	NA	NA	0.36	< 0.001	1.40	Fatty acids	C16537
Phenylalanine	0.74	0.004	1.01	NA	NA	NA	0.69	< 0.001	1.12	Amino acids	C00079
Pyroglutamic acid	0.58	< 0.001	1.33	NA	NA	NA	0.54	< 0.001	1.42	Amino acids	C01879
Pyruvic acid	NA	NA	NA	0.59	0.002	2.07	2.00	< 0.001	1.51	Organic acids	C00022
Ricinelaidic acid	NA	NA	NA	4.52	0.009	1.77	NA	NA	NA	Fatty acids	C08365
Ricinoleic acid	NA	NA	NA	2.35	< 0.001	2.02	0.21	< 0.001	1.50	Fatty acids	C08365
Sebacic acid	NA	NA	NA	NA	NA	NA	0.81	< 0.001	1.19	Fatty acids	C08277
Stearic acid	0.40	< 0.001	1.92	NA	NA	NA	0.33	< 0.001	1.71	Fatty acids	C01530
Suberic acid	0.55	< 0.001	1.18	NA	NA	NA	0.47	< 0.001	1.40	Fatty acids	C08278
Tridecanoic acid	0.77	< 0.001	1.33	NA	NA	NA	0.70	< 0.001	1.61	Fatty acids	C17076
Tryptophan	0.61	< 0.001	1.57	NA	NA	NA	0.66	< 0.001	1.31	Amino acids	C00078
Tyrosine	0.63	< 0.001	1.46	NA	NA	NA	0.56	< 0.001	1.35	Amino acids	C00082
Undecanoic acid	0.85	0.004	1.29	NA	NA	NA	0.79	< 0.001	1.45	Fatty acids	C17715

Abbreviations: ABS, acute abstinence syndrome; DHA, docosahexaenoic acid; DPA, docosapentaenoic acid; FC, fold change; FDR, false discovery rate; HCs, healthy controls; KEGG, Kyoto Encyclopedia of Genes and Genomes; NA, not available; PABS, postabstinence syndrome; SCFAs, short-chain fatty acids; VIP, variable importance in the projection.
